# The Challenge of Single-Photon Emission Computed Tomography Image Segmentation in the Internal Dosimetry of ^177^Lu Molecular Therapies

**DOI:** 10.3390/jimaging10010027

**Published:** 2024-01-19

**Authors:** Joanna Gawel, Zbigniew Rogulski

**Affiliations:** Faculty of Chemistry, University of Warsaw, 02-093 Warsaw, Poland

**Keywords:** segmentation, SPECT, ^177^Lu therapy, threshold segmentation, region growing, CNN, fuzzy C-means

## Abstract

The aim of this article is to review the single photon emission computed tomography (SPECT) segmentation methods used in patient-specific dosimetry of ^177^Lu molecular therapy. Notably, ^177^Lu-labelled radiopharmaceuticals are currently used in molecular therapy of metastatic neuroendocrine tumours (ligands for somatostatin receptors) and metastatic prostate adenocarcinomas (PSMA ligands). The proper segmentation of the organs at risk and tumours in targeted radionuclide therapy is an important part of the optimisation process of internal patient dosimetry in this kind of therapy. Because this is the first step in dosimetry assessments, on which further dose calculations are based, it is important to know the level of uncertainty that is associated with this part of the analysis. However, the robust quantification of SPECT images, which would ensure accurate dosimetry assessments, is very hard to achieve due to the intrinsic features of this device. In this article, papers on this topic were collected and reviewed to weigh up the advantages and disadvantages of the segmentation methods used in clinical practice. Degrading factors of SPECT images were also studied to assess their impact on the quantification of ^177^Lu therapy images. Our review of the recent literature gives an insight into this important topic. However, based on the PubMed and IEEE databases, only a few papers investigating segmentation methods in ^177^Lumolecular therapy were found. Although segmentation is an important step in internal dose calculations, this subject has been relatively lightly investigated for SPECT systems. This is mostly due to the inner features of SPECT. What is more, even when studies are conducted, they usually utilise the diagnostic radionuclide ^99m^Tc and not a therapeutic one like ^177^Lu, which could be of concern regarding SPECT camera performance and its overall outcome on dosimetry.

## 1. Introduction

When we consider the risk–benefit ratio of a given application of radiation, the benefits outweigh the risks in the application of radiopharmaceutical diagnostics in which assessments are conducted for a given population. However, this does not apply to therapy, in which toxicity and accuracy are important and patient-specific, and dosimetry is justified to plan treatment. In the case of radionuclide therapy, nuclear images are collected and used to optimise treatment and assess responses. To obtain information about the potential detriment to organs, as well as the doses accumulated in tumour lesions, these tissues must be properly segmented in the collected images. 

The concept of the mean absorbed dose, along with the assumption of uniform dose distribution within a given volume, as widely used in internal dosimetry, are assumed to comprise predicting factors for biological effects. This can be assumed to be true for stochastic effects, like cancer induction. However, in the case of deterministic effects (tumour control and normal tissue toxicity), nonuniform doses and dose rates may cause overdosing in certain parts but underdosing in others, leading to treatment failure and leaving some clonogenic cells with sublethal doses [1]. The biologically effective dose (BED) concept that is widely used in external beam radiotherapy and brachytherapy has been extended and incorporated into the Committee on Medical Internal Radiation Dose (MIRD) schema to be applied to organs at risk, like kidneys and bone marrow. BED takes into account the total absorbed dose, the absorbed dose rate, and the effects of repair and fractionation; it is specific to a tissue and a given radiobiologic endpoint for which a linear quadratic model is applied to obtain the α/β ratio [2]. This makes tissue segmentation even more important if we want to optimise the treatment on the voxel level in terms of possible under- and over-dosing.

## 2. Background Information

### 2.1. ^177^Lu Characteristics

Recently, there has been increasing interest in the use of ^177^Lu as a theranostic radionuclide due to its physical and chemical properties. ^177^Lu is a β^−^ emitter with a half-life of 6647 days, that decays to stable 1^77^Hf. During this disintegration, it emits electrons (β^−^ particles and internal conversion (IC) electrons) with a mean kinetic energy of 147 keV and β^−^ maximum energies with abundances as follows: E_β max_ = 497 keV (78.6%), 384 keV (9.1%), and 176 keV (12.2%). For electrons with these energies, their ranges (CSDA—continuous slowing-down approximation) in the unit density soft tissue are as follows: 0.28 mm for mean kinetic energy and 1.8 mm for maximum electron energy [3,4,5]. These ranges are very convenient for therapeutic purposes because of their sparing effect on healthy tissue. Because ^177^Lu also has a gamma ray spectrum of good abundance and energies, making it suitable for imaging with single photon emission computed tomography (SPECT), it may be used to obtain pharmacokinetic and pharmacodynamic data for ^177^Lu-labelled radiopharmaceuticals. It is also possible to estimate absorbed doses for tumours and organs at risk [4]. 

The chemistry of ^177^Lu allows for the creation of stable products that have good in vivo characteristics. Lutetium is the last of the lanthanide series; it has 71 electrons in a [Xe]4f 145d16s2 configuration. It most often occurs in the +3 oxidation state, which is characteristic of the lanthanides. Lutetium has the lowest coordination number of all the lanthanides, which results from the fact that this element has a fully electron-filled 4f orbital, while the s, p, and d orbitals remain empty, so the ionic radius of Lu3^+^ is the smallest of all the lanthanides; this affects the possibility of ligand binding. In solution, these cations have a strong tendency to form complexes with atoms that are strong electron donors, such as O, F^−^, or N. The coordination number is usually 8 or 9, and the resulting complexes with acrylic ligands or cyclic polyaminopolycarboxylates with eight or nine donor atoms are usually very thermodynamically stable. DOTA (1,4,7,10-tetraazacyclododecane-1,4,7,10-tetraacetic acid) is one such ligand, of which the complex with ^177^Lu shows a high thermodynamic stability and kinetic inertia [4].

At present, there are two main therapeutic applications of radiopharmaceuticals labelled with ^177^Lu: the treatment of metastatic neuroendocrine tumours with the use of somatostatin receptors, and the treatment of metastatic castration-resistant prostate cancer with the use of PSMA-targeting ligands [3].

### 2.2. ^177^Lu Therapy of Neuroendocrine Tumours

Gastro-entero-pancreatic neuroendocrine (GEP/NET) tumours are a subtype of well-differentiated neuroendocrine tumours (NET) that arise from the diffuse endocrine system. GEP/NETs are rare tumours, with an incidence level of 1.8/100,000/year in Europe. However, because they are also low-grade tumours (1–3 grade based on Ki67 and mitotic index), there are many patients living with the disease (with an estimated prevalence of 38/100,000 in Europe) [6]. For patients with localised disease, the primary treatment is usually surgery with curative intent, while for unresectable widely metastatic and symptomatic disease, systemic treatment with somatostatin analogues and/or interferon-α is required, because external beam radiotherapy and chemotherapy are considered to be ineffective in such cases [7,8]. However, diagnosis in the early stages can be challenging, because patients are usually asymptomatic until they are metastatic [7]. Metastatic disease is observed in 30% of patients at diagnosis [6]. 

GEP/NET tumours overexpress somatostatin receptors, in particular subtype 2 SSTR_2_, a feature that is used in both diagnosis and treatment. In diagnostics, octreotide, that mimics somatostatin, is bound with high affinity to these receptors, and after labelling with radionuclides like ^111^In or ^123^I, it is used in scintigraphy as a very sensitive diagnostic method for the detection of primary tumours and metastases [8]. In therapy, the same receptor biding somatostatin analogue (SSA), Tyr(3)-octreotate (TATE), is labelled with ^177^Lu or ^90^Y with the use of DOTA chelator for therapeutic purposes [6,8]. This allows us to selectively deliver radiation to tumour cells that overexpress SSRT_2_ receptors. ^177^Lu is the preferred radionuclide for these purposes due to its ability to spare healthy tissue, because it has shorter path length (2 mm in tissue compared to 12 mm) than ^90^Y as a result of its lower beta emission energy (147 keV compared to 934 keV, respectively). In particular, for radiosensitive glomeruli, it lowers the risk of developing nephrotoxicity [6]. This radionuclide is feasible for therapeutic purposes, not only because of its energy range, but also due to its other characteristics, like its physical half-life (6.67 days) and the abundance of emitted β^−^ particles and gamma photons, which makes it possible to perform imaging with SPECT emission tomography and dosimetry [4]. 

Tumour-targeted peptide receptor radionuclide therapy (PRRT) for GEP/NETs expressing somatostatin receptors was approved by the EMA in 2017 and by the FDA in 2018 for clinical use as Lutathera^®^ in the management of patients with unresectable and metastasised neuroendocrine tumours. The standard treatment consists of four solution infusions at a dose of 7.4 GBq, 8 weeks apart (cumulative activity 29.6 GBq [800 mCi]), unless unacceptable toxic effects occur [9,10].

### 2.3. Lu^177^ -PSMA in the Treatment of Castration-Resistant Prostate Cancer

Currently, the most widely used product of the gene expression characteristic of the prostate cancer process is PSMA (prostate-specific membrane antigen). It is an antigen of the cell membrane of prostate cells, a type II transmembrane glycoprotein. Due to its enzymatic properties, it is expressed up to a thousand-fold in poorly differentiated metastatic and castration-resistant prostate cancer cells [11]. PSMA receptors enable the internalisation of surface-bound proteins into endosomes through the process of endocytosis. The use of this phenomenon makes it possible to introduce a radioisotope into tumour cells [12].

There is a correlation between PSMA expression and tumour grade [13,14]. It has been noted that the expression of PSMA seems to be inversely proportional to the level of androgens. This is important when a patient is undergoing hormone therapy during which the level of androgens is lowered [13,15]; additionally, after androgen therapy, the expression of PSMA in cancer tissue and cells increases [15]. As was shown, the expression of PSMA in metastatic tissue is very high, making it possible to use of this phenomenon in the targeted therapy of cancer recurrence after androgen therapy [15,16].

Because the expression of this receptor is highly dependent on the stage of the tumour, it is possible to link it with the Gleason scale; thus, PSMA is a very good target for radionuclide therapy. Analyses also showed the possibility of using PSMA expression as an independent predictive measure to assess the probability of biological recurrence [17]. However, PSMA is not a fully specific receptor for prostate cancer, and its expression is also found in other regions, such as the small intestine, kidneys, or salivary glands [11]. This will have an impact on the ultimate toxicity of radionuclide therapy, also causing dose accumulation in these areas, which affects the width of the therapeutic window for this type of therapy.

PSMA ligands developed for diagnostic and therapeutic applications in prostate cancer were originally based on 2-[3-(1,3-dicarboxypropyl)ureido]pentanedioic acid (DUPA) [18]. PSMA-11, on the other hand, was developed on the basis of a gallium-specific chelator: N,N’-bis(2-hydroxy-5-(ethylene-beta-carboxy)benzyl)ethylenediamine N,N’-diacetic acid (HBED-CC) [19]. This chelator transpired to be more efficient in this case than the widely used DOTA; however, coordination is only possible with ^68^Ga. To bind other trivalent metals such as ^177^Lu or ^111^In, based on DOTA, PSMA-617 was created [20]; this has opened the way for therapeutic applications. Currently, there are several PSMA ligands in the form of peptides and antibodies labelled with ^177^Lu, either in the phase of clinical trials or already being clinically used in the treatment of castration-resistant prostate cancer metastases [11].

### 2.4. Radiobiological Consideration

In the case of therapy, not only the stochastic effects of ionising radiation are of concern, but, above all, the deterministic ones. The dose absorbed by normal tissues may be significant, which may lead to both an increased risk of secondary malignancy (increase in stochastic risk) and deterministic effects such as haematological toxicity, myelosuppression, or fibrosis. For peptide receptor radionuclide therapy (PRRT) with the use of somatostatin receptors, the dose limiting factor is the sensitivity of the bone marrow to radiation, because the somatostatin receptors are expressed on stem cells [21]. An absorbed dose to bone marrow below 2 Gy is assumed to be safe to avoid hypoplasia. For an absorbed dose of 2 Gy to bone marrow, the probability that the patient develops leukaemia is approximately 2% [21]. Also, nephrotoxicity can be a serious adverse effect. Other endpoints, as was shown in the NETTER-1 trial, include the occurrence of gastrointestinal toxicity (18 patients (17%) developed grade 3 or 4 adverse events), thrombocytopenia, and lymphopenia (grade 3 or 4 blood disorders observed in 14 patients (13%)) in the treatment group [6,9]. These effects can be partially avoided by blocking the organs with appropriate drugs. For example, ^177^Lu-DOTATATE is excreted through glomerular filtration in kidneys, but its reabsorption by proximal tubules increases radiation exposure. To spare the kidneys and avoid nephrotoxicity, intravenous infusion of positively charged amino acids lysine and arginine is applied to block the receptors and decrease the binding of ^177^Lu-DOTATATE in proximal tubules [6]. However, to improve treatment outcomes, a more robust methodology is required. SPECT/CT-based 3D dosimetry for individual patients with the use of regional or even voxel-based calculations and dose-volume histograms obtained before treatment may help to reduce dose calculation errors and spare healthy tissue, as is already the case in conventional external beam radiotherapy. 

In conventional external beam radiotherapy, as well as in brachytherapy, the quantity used for local tumour control is the dose absorbed in the target volume. This makes it possible to predict the radiobiological response to treatment [22]. Assessments of radiation doses absorbed in critical organs make it possible to determine the likelihood of adverse effects, i.e., they allow for the estimation of treatment toxicity. However, therapy with the use of radiopharmaceuticals is still treated as a type of chemotherapy, with strictly defined activity values administered to patients, differentiated only by the patient’s weight or body surface area. Research shows [23], however, that this approach often leads to ineffective treatment. Considering inter-patient differences in pharmacokinetics, radionuclide therapy might be improved by applying an individual dosimetry approach [24]. Individual dosimetry can bring great benefits to patients, and it is in accordance with the currently applicable Basic Safety Standards (BSS) directive. Additionally, the need for individual dose planning in the target volume and assessment of the exposure of normal tissues also applies to therapy using radioisotopes [6,21,25].

### 2.5. Absorbed Dose Calculation

The Medical Internal Radiation Dose (MIRD) protocol defines the framework conditions for the assessment of the absorbed dose, which comes from the source region into the given region, called the target. The absorbed dose (*D*[*r_T_*]) is calculated as the product of cumulative activity over time and the so-called the S-value, according to Equation (1), expressed in greys (Gy). The source or target regions may be any well-defined tissue volume, organ, or the whole body, but also a voxel, cell, or cellular structure.

(1) $$D\left(r_{T}\right)=\tilde{A}(r_{S})•S(r_{T}\leftarrow r_{S}\;)$$

where the source region is denoted by *r_S_* and the target region by *r_T_*, $\tilde{A}(r_{S})$, i.e., cumulative activity, is the number of decays that take place in a given region in a specific period of time, and *S* is the absorbed dose rate per unit volume [Gy/s/Bq].

Cumulative activity $\tilde{A}$ (*r_S_*, t), i.e., the number of decays in the source area, is calculated as the area under the curve defining the decrease in activity over the time in the source area after administration of the radiopharmaceutical; see Equation (2). It can be obtained on the basis of images collected in subsequent acquisitions. It can also be determined by direct tissue measurements (from a biopsy), blood sample measurements, or whole-body measurement of activity with a gamma ray probe. A theoretical method that can be used to predict activity in a source region for which measurements are impossible is compartmental modelling.

(2) $$\tilde{A}\left(r_{S},T_{D}\right)=\int_{0}^{T_{D}}A\;(r_{S\;},t)dt$$

The time interval for calculations is from the time of administration to the biologically effective endpoint, *T_D_*. The residence time, which is the time the activity is present in the source area, may be calculated as follows:

(3) $$\tilde{a}=\frac{\tilde{A}(r_{S})}{A_{0}}$$

where *A*_0_ is the activity at the time of administration.

Each source region is identified, and its uptake over time and retention time are assessed. These are the inputs. Any high uptake region should be considered as a source region. Also, the rest of the body, as a sum, should be considered as a potential source region. Where it is not possible to obtain data from direct measurements, compartmental modelling can be used to predict the behaviour of a given factor in a given area. The total average dose contributing to the target area is obtained by summing the contributions from the individual source areas:

(4) $$D\left(r_{T},\;T_{D}\right)=\sum_{r_{S}}\int_{0}^{T_{D}}A\left(r_{S},t\right)S\left(\left(r_{T}\leftarrow r_{S}\right),t\right)dt$$

The dose rate values per unit of activity (so-called *S*-values) are generated using Monte Carlo simulations in the digital anatomic model [26].

### 2.6. Calibration of the SPECT System

The camera system must be well calibrated to obtain quantitative distribution of accumulated activity for the absorbed dose calculations. Conversion from a voxel value to activity is carried out with the use of a calibration factor. There are two widely accepted approaches to camera calibration [1]; the first is the use of a calibration point source of ^177^Lu along with the properly calibrated activity meter. This methodology assumes that the activity meter is calibrated for ^177^Lu in a Secondary Standards Dosimetry Laboratory (SSDL) that allows traceability to be maintained, so that the result can be related to an appropriate standard. It is also important to ensure the same calibration and source measurement geometry (the same type of vial and volume of measured solution) [27,28]. The point source placed in air is imaged using planar acquisition. The camera calibration factor is then obtained by dividing the total counts in the region of interest (ROI) that surrounds the point source by the acquisition time and activity [28]. However, this approach assumes that highly accurate correction for scatter and attenuation is in place; otherwise, the outcome will be biased. This approach is especially important when calculating the calibration factor for ^177^Lu, where high-energy scattered photons may have an impact on the total counts in ROI, which must be carefully corrected with the use of, e.g., the triple-energy window (TEW) technique [1]. The second approach, which is considered more reliable, is performed with a torso phantom, filled with water, and well calibrated ^177^Lu sources which are imaged with the clinically used SPECT protocol and reconstruction methods [1,28]. The geometry obtained in such a methodology approximates the scatter and attenuation properties of patient studies much better and has been suggested by the EANM/MIRD Committee as the method of choice for internal dosimetry, especially in ^177^Lu therapies [1,28]. 

#### Image Acquisition

The overall performance of gamma cameras is of great importance in quantitative studies that lead to dose calculations in radionuclide therapies, because it may strongly compromise the outcomes if it has not been well optimised [29]. The trade-off that is needed between high system sensitivity and spatial resolution is even more pronounced for the higher energy of photons and multiple emissions typically associated with therapeutic radionuclides like ^177^Lu. In Monte Carlo simulations, two thicknesses of NaI(Tl) crystal were investigated along with four types of collimators, to assess system sensitivity, with 20% energy windows centred on 113 keV and 208 keV photopeaks [28,30]. Low-energy high-resolution (LEHR) and low-energy general-purpose (LEGP) collimators are better, if the system sensitivity is considered, than medium-energy (ME) or high-energy (HE) collimators. However, ME collimators that yield lower septal penetration may be used in therapy with ^177^Lu, which emits high-energy photons [28]. When the crystal thickness is considered, for a 208 keV photopeak, better sensitivity results were obtained with a crystal of 5/8″ thickness than 3/8″ thickness. This results in better statistical noise reduction. Conversely, for a 113 keV energy photopeak, a 3/8″ crystal showed better results for sensitivity measurements because of the contribution of 208 keV photon backscattering in the material behind the crystal (the 180° backscatter photon energy is 114.6 keV) to the image counts. When, as suggested, an ME collimator is used, the energy window should be centred at 208 keV with a 15–20% energy window. If the number of counts is not sufficient to obtain optimal image quality, a second window with a 113 keV photopeak could be considered. For each dataset, attenuation and scatter correction should be performed separately before merging them for further analysis [28]. Extended analysis concerning the energy window and collimator properties can be found in [1,28,30]. Additionally, the use of 128 × 128 projection matrices is suggested for ^177^Lu acquisitions if the counting rate is sufficient, and 60–120° projection angles with AutoContour orbits to improve image resolution [28]. 

The conversion of gamma ray energy into an electronic signal and its processing requires a certain amount of time, called dead time. If the event rate is too high, individual events are not counted correctly, resulting in count losses. These losses must be corrected. This is particularly important in therapy application when high levels of activity are required. ^177^Lu, however, has a low yield of γ-photons and a small bremsstrahlung contribution; thus, dead time corrections are required mainly in projections acquired shortly after injection (this could affect the first time point of the time-activity curve) [28,31]. Dead time correction can be assessed on the basis of phantom studies that use a clinical acquisition protocol and scatter correction.

### 2.7. SPECT Image Degradation Factors

There are three main factors that degrade the image quality in emission tomography: photon attenuation, Compton scattering, and the collimator–detector response characteristics. Attenuation and collimator–detector response (CDR) compensations are usually performed by directly including certain correction factors in the system–transition matrix. In the case of scattering, the situation is more difficult, because the scatter distribution is a complex function. Thus, including a precalculated scatter estimate in the iterative algorithm is a better approach to avoid the requirement of extensive computations [1,32]. 

#### 2.7.1. Photon Attenuation

Because the patient’s tissue composition is heterogenous, emission data must be corrected in an accurate, patient-specific manner to obtain high-quality quantitative data. Proper attenuation correction requires the creation of an attenuation map that contains the spatial distributions of the linear attenuation coefficients (µ) of the SPECT photon energy. Such an attenuation map is then incorporated into the reconstruction algorithm to correct the emission data [33]. Although such maps can be obtained with the use of a radionuclide source, CT-based attenuation correction is considered to be the method of choice nowadays. CT correction is characterised by lower noise, greater spatial resolution, and better contrast conditions than the radionuclide source technique [1]. What is more, the linear attenuation coefficient strongly depends on the density of the medium; to improve the accuracy of image quantification, it is strongly recommended that CT correction maps be used in internal dosimetry applications [28]. A CT attenuation map is generated on the basis of a CT scan (usually with a matrix size of 512 × 512) that is down sampled to fit the SPECT image voxel size and slice thickness (128 × 128 matrix size) and spatially aligned with the SPECT image set. Then, a translation from Hounsfield units (HU) to the corresponding linear attenuation coefficients for ^177^Lu photopeak energy is performed. Usually, it is a voxel-by-voxel scaling method that does not require image segmentation [1]. Linear attenuation coefficients are also energy dependent, and CT values for X-ray energies must be scaled for the SPECT radionuclide photon energy. Such calibrations are usually made with the use of a phantom containing inserts of materials of known composition [1].

#### 2.7.2. Compton Scattering

One of the main image degradation sources in SPECT is Compton scattering, which can happen anywhere in the patient or surrounding material, i.e., the table, collimator, or other camera parts. Scattered photons can originate from higher-energy down-scattering or self-scattering processes. Several methods for scatter compensation are mentioned in the literature. They usually fall into one of two categories: energy-distribution-based methods and spatial-distribution-based methods. However, only a few of them have found their place in clinical applications, i.e., dual-energy window (DEW) or triple-energy window (TEW) correction and effective scatter source estimation (ESSE) [1].

In the TEW method [34], the number of scattered photons in the photopeak is estimated on the basis of the number of counts in the adjacent scatter windows set on both sides of the photopeak window. This method accounts for both self-scattered photons and down-scattered ones. The scatter fraction in the photopeak window can be calculated as follows:

(5) $${SE}_{pw\;}=\left(\frac{C_{lw}}{W_{lw}}+\frac{C_{uw}}{W_{uw}}\right)\times \frac{W_{pw}}{2}$$

where *C_lw_* and *C_uw_* are pixel counts in the lower and upper windows, and *W_lw_*, *W_uw_*, and *W_pw_* are the lower, upper, and photopeak window widths, respectively [28]. The scatter estimate is further applied into the projection step of the iterative reconstruction algorithm. For ^177^Lu studies, MIRD pamphlet no. 26 suggests the application of the TEW method with the use of LE collimators and a 113 keV photopeak or ME collimator for a 208 keV photopeak only or an ME collimator for both photopeaks [28]. However, later research [35,36,37] with Monte Carlo simulations revealed that the use of the TEW technique in ^177^Lu therapy applications may lead to under- or over-estimates of activity and should be applied with caution. What is more, the TEW method is noisy because of the narrow window used; thus, noise reduction and low-pass filtering must be used. A wider scatter window can reduce noise; however, this also results in poorer accuracy in the scatter approximation, so optimisation with a Monte Carlo simulation is needed [36]. 

De Nijs et al. suggested using both energy photopeaks and an ME collimator with the ESSE technique [38]. Uribe et al. recommended the analytical photon distribution interpolated (APDI) method for scatter correction for lesions in non-uniform attenuation areas [4]. The ESSE method uses precalculated kernels obtained from MC simulation for self-scatter estimations, while APDI analytically calculates scatter distribution with the use of the Klein-Nishina cross-section for Compton scattering [28]. 

Andrew P Robinson et al. suggested that even with TEW correction localised and optimised for ^177^Lu, it is not possible to reflect patient-specific activity distribution with good accuracy because of the highly complex nature of the Compton scattering process. The problem might be fully addressed with the use of a Monte Carlo simulation, which has the potential to provide a corrected image with reduced uncertainty [36].

#### 2.7.3. Collimator–Detector Response (CDR)

Detector–collimator response is a metric that corresponds to the image generated by the system in response to a point-like source. The shape of the CDR is the main factor that determines the image resolution in SPECT. It comprises four components, i.e., the intrinsic response of the detector and the collimator response, which, in turn, comprises three components: geometric, septal penetration, and scatter [39]. The intrinsic spatial resolution is modelled with a Gaussian function, and for a modern system, it is usually a value of approximately 4 mm or less at full width at half of the maximum (FWHM) of the point spread function (PSF). However, the extrinsic resolution (with the collimator on) varies greatly with the source-to-collimator distance, and for ME collimators, its typical value at 10 cm is around 10 mm. To account for the significant effects of septal penetration and scatter components, Monte Carlo simulations, along with an appropriate experiment, can provide necessary information for the compensation function. Reconstruction algorithms that employ CDR compensation improve the spatial resolution and change the noise texture to smooth the image. CDR compensation may, however, produce Gibbs artefacts in the areas near the sharp boundaries and change the number of counts in voxels [28]. 

### 2.8. Partial Volume Effect (PVE)

Because SPECT systems have limited spatial resolution, activity information is lost for small objects or at the edges of structures. The effect is called the partial volume effect; it is apparent for small structures (smaller than 3× FWHM of spatial resolution) and is caused by the spill-in and spill-out effects on the edges of the structures. If the activity outside the volume of interest (VOI) is higher than that in the structure, the spill-in effect means that the activity from outside the structure is integrated into the volume of interest and there is an artificial increase of the overall activity in the VOI. For the opposite situation, where the activity in the structure is higher than the background activity, the spill-out affects assessments of underlying activity. All of this blurs the boundaries of the regions, and thus, exact information about activity localisation becomes difficult to obtain [1,40]. To overcome this problem, recovery coefficients (RC) are usually determined on the basis of a series of phantom studies, where RC is the ratio between the derived image and the true activity. In phantom experiments, a series of spherical test objects of known sizes are usually placed inside the phantom. Because the PVE is strongly affected by structure size and shape, as well as the data acquisition protocol and reconstruction method, it is important to acquire and process images for the recovery curve in the same manner as that applied to patient data. The same clinical protocol and reconstruction algorithms should be used to reflect the anatomic structures of the patient. However, as was shown in recent studies, this approach might be too simplistic to assess the PVE with good accuracy [38,40]. In a series of 3D-printed kidney phantom studies, Grings et al. demonstrated that PVE is not only strongly affected by the specific shape of the kidney for a certain patient, but also that its magnitude depends on the intra-renal activity distribution. Additionally, it was shown that using the ratio of surface area-to-volume of phantoms as an indicator is a convenient approach to modelling a kidney [40]. 

Nevertheless, work is ongoing, and some interesting solutions, like automatic atlas-based Partial Volume Corrections (PVC), have been proposed to overcome the PVE and improve the quality of SPECT images [41]. In addition to molecular imaging, the method requires contrast-enhanced computed tomography and manual segmentation of many organs. However, the results show that it is practical and comparable to manual segmentation. What is more, with an increasing number of atlas images, this method has the potential advantage of facilitating SPECT image segmentation. Another approach is multimodal image segmentation in which, for example, MRI images are used to reduce the PVE on PET images [42]. 

### 2.9. SPECT Image Reconstruction

Attenuation, scatter, and collimator-detector response (CDR) can be compensated for in a reconstruction by modelling their effects in the process of the iterative reconstruction [1,28]. Iterative reconstruction methods like ordered-subset expectation maximisation (OSEM) are recommended for therapeutic purposes. The number of iterations could be optimised with regard to the complexity of the problem and the compensations that are being made during reconstruction in order to determine the degree of convergence from the required accuracy. MIRD Pamphlet No. 23 assumes a recovery of 90% as a convergence, where recovery is the ratio of activity estimated from the image to the true activity in the object [1]. If the mean absorbed dose is to be calculated, a larger number of iterations may be considered to compensate for image-degrading factors and to reduce noise and edge artefacts by averaging them with an increasing number of iterations. However, when voxel-based dosimetry is considered, the better approach is to reduce the number of iterations to preserve the image voxel information, because the noise tends to be amplified with an increasing number of iterations. Additionally, when CDR compensation is used, it may generate some edge artefacts (Gibbs like) that also become more pronounced with an increasing number of iterations [1,28]. If the need for noise reduction is considerable, low-pass filtering may be used if the total counts in the data after filtering are preserved [28]. 

It needs to be underlined, however, that the greater the smoothing, the more difficult it is to segment structures due to the fact that an image with greater smoothing has a smaller absolute range of intensities. Additionally, higher thresholding is required to compensate for the decrease in contrast [43].

## 3. Standard Segmentation Approaches

The purpose of the segmentation is to divide the image into non-overlapping areas. Segmented areas correspond, by definition, to physical objects of fixed characteristics that are appropriate for the intended medical purpose. When segmented areas are known, further analysis might be implemented. The criteria used for segmentation can be subjective when the radiologist is contouring regions in a medical image, or objective when the process of segmentation is performed automatically with the use of segmentation algorithms. Regardless of the method used, the quality of the segmentation process outcome has a significant impact on the medical diagnosis or therapy in the case of internal dosimetry. 

The European Association of Nuclear Medicine Dosimetry Committee has suggested, for dosimetry purposes, that at risk organs should be segmented on SPECT/CT images using CT information and with the applied correction for organ movements (adjustment to SPECT datasets). In the case of tumour delineation, this can be challenging with low-contrast CT images, especially without contrasting agents, and so segmentation of SPECT images is suggested [3]. It is also important to underline that tumour volumes, as delineated on diagnostic functional images with the use of different radionuclides, may not be the same as the volume obtained with a therapeutic radionuclide like ^177^Lu, due to the different physiology [1]. 

Generally, in the case of rapidly shrinking tumours, which may respond dramatically to treatment (within days), such as malignant lymphomas, it is advisable to redefine the organ and tumour volumes in each of the register series of images [1]. However, this is not the case for NETs, and a single volume of interest could be segmented in the reference scan and then applied to the rest of the scan series unless they are not properly registered to the reference scan. 

### 3.1. Evaluation Metrics and Validation

#### 3.1.1. Gold standard—Manual Delineation by a Skilled Operator

Manual segmentation by a skilled operator, to this day, is the gold standard to which other segmentation methods for the delineation of tumour lesions and organs may be compared. To perform this task properly, however, one needs to collect manual segmentations from as many different operators as possible, to evaluate inter-observer reliability, and to obtain as many manual segmentations from each operator as possible for intra-observer reliability. To obtain a single, valuable evaluation, i.e., the so-called ground truth, the above-mentioned segmentations should be statistically combined. In the proposed methodologies, the judgement of trueness of a voxel belonging to a structure is made on the basis of majority agreement among experts. For the performance evaluation of a given segmenting algorithm with respect to the ground truth, a contingency table is produced with true positive (TP), true negative (TN), false positive (FP), and false negative (FN), where a pixel belonging to the structure of interest is considered positive and one belonging to the background is considered negative [44]. These values make it possible to derive performance metrics like accuracy, sensitivity, and specificity. 

There are three different categories of segmentation assessments after the ground truth has been established: quantification of volumetric differences with the use of estimators derived from a confusion matrix; shape-based similarity measures; and statistical methods based on regression [43,45]. The simplest method, often used in clinical practice, is the absolute percentage difference in the total volume of two segmented structures. Although commonly used, this approach is not enough to determine the similarity of segmentations if it is the only metric applied, because the segmentation method under investigation could have the same volume as the ground truth while having a completely different shape. Quantitative metrics with statistical estimators should be used for more reliable assessments.

#### 3.1.2. Dice Similarity Coefficient

To combine statistical estimators into one single value, the Dice similarity coefficient was created. This index is computed as the ratio of the number of elements in the intersection of two volumes to the mean of those volumes [45]. The two volumes are the segmented volume of interest (*A*) and the ground truth volume (*B*).

(6) $$DSC\;(A,B)=2\frac{\left|A\cap B\right|}{\left|A\right|+|B|}$$

##### DSC Is Also Known as the F_1_ Score

Based on the confusion matrix, measures like accuracy, sensitivity, and specificity can be derived as follows:

(7) $$\mathrm{Accuracy}:\;\frac{TP+TN}{n}$$

(8) $$\mathrm{Sensitivity}:\;\frac{TP}{TP+FN}$$

(9) $$\mathrm{Specificity}:\;\frac{TN}{TN+FP}$$

Sensitivity, also called the true positive rate, is the probability that an actual positive will give a positive test outcome, while specificity, or the true negative rate, is the probability that an actual negative will give a negative test outcome. In addition to the commonly used metrics, there are some additional ones that can be applied, such as the false positive rate, positive predictive value (PPV), and the negative predictive value (NPV). PPV provides information about the likelihood that a given pixel classified as positive is really part of this structure. Conversely, NPV gives information that the pixel does not belong to the structure when it is classified as negative. 

#### 3.1.3. Boundary-Based Measures 

The Hausdorff distance is a kind of overlapping index that measures how far two segmentations under investigation are from each other. This measure is recommended for complex boundaries and thin structures, such as cerebral blood vessels. Because it also takes into account the location of the pixel, it provides an advantage in comparison to other metrics, like DSC [46]. The Hausdorff distance and Dice similarity coefficient are usually both reported for more robust segmentation performance assessments. 

#### 3.1.4. Statistical Methods—Spearman and Pearson Coefficients

Regression-based measures like the Spearman and Pearson coefficients, as well as mean volume difference and the relative volume ratio, are often used in the clinical literature. However, it must be underlined that without metrics like DSC, sensitivity or specificity, such an approach to assessing segmentation performance lacks information about its accuracy [43]. 

## 4. Threshold-Based Methods

### 4.1. Phantoms Used in Assessments

Phantoms are well established gold standards in nuclear medicine assessments that also concern internal dosimetry. However, as demonstrated in some studies, they are sometimes overly simplistic [40]. Evaluations with such a simple model can be heavily biased and lead to over- or under-estimates of tissue activity and potentially also absorbed doses. Such estimates may result in poor therapeutic outcomes. Nevertheless, phantoms play a very important role in the evaluation and comparison of different segmentation algorithms. They provide a very useful tool for basic validation and training for such algorithms, serving as a gold standard that contains structures of known features [45]. However, validation and further assessments on real clinical images, which are far more complex, are needed. In the studies under investigation, several different phantoms were utilised. 

Phantom-based assessments are used to estimate the performance accuracy of imaging methods [47]. Parameters such as system sensitivity and dead-time corrections, and problems such as appropriate correction methods and the accuracy of image quantification with different collimators and radionuclide energies, are determined in experiments using physical phantoms. These phantoms are usually cylinders filled with water or background activity, inside which spherical or cylindrical objects filled with different ^177^Lu activity concentrations are placed. However, due to the complexity of human anatomy, they are usually not sufficient to assess the accuracy of segmentation methods. To overcome this, digital phantoms can be constructed, like synthetic images. The extended cardiac-torso (XCAT) family is an example of digital phantoms that can be coupled with the ^177^Lu-DOTATATE pharmacokinetic model. In experiments by Gustafsson et al., phantoms including two or three tumours of various ranges obtained from patient SPECT images were used. Additionally, with the help of the Monte Carlo code SIMIND, SPECT projections were also simulated to conduct the experiments [48,49]. 

### 4.2. Fixed Thresholding Methods

Thresholding is one of the simplest methods of image segmentation; it compares the intensity in each pixel with a fixed threshold value. The method is based on grey-level histograms. If the intensity is higher than the threshold value, the pixel is given a value of 1 (sometimes 255), otherwise 0. The outcome is a binary image whose pixels have only two possible intensity values, usually black or white. Then, areas with the same characteristics (e.g., only white) are searched for in the image, and regions are connected to obtain a final image with a segmented structure. Because the resulting images usually have jagged edges, contain holes, and the segmented areas do not necessarily correspond to the physical structure, postprocessing is usually needed [50]. The threshold is either derived from the basis of maximum count, where it constitutes a fixed percentage of that value, or from a statistical methodology.

As was found by Erdi et al. in phantom studies, a threshold of 42% of the maximum voxel intensity in initialisation ROI resulted in minimal segmentation error (for spheres equal to or larger than 20 mL (diameter D > 38 mm) and target–nontarget ratios higher than 5:1); this threshold is often used in SPECT segmentation [51]. Although the fixed threshold method is simple, the appropriate threshold level strongly depends on the signal-to-background ratio [52]. What is more, a single value might not be sufficient for every clinically relevant structure in the image [53]. This method is also strongly biased by the acquisition and reconstruction algorithms. To address these issues, some adaptations of the aforementioned methodology were developed to incorporate the effects of background and target size.

Statistical estimates of the threshold are based on the minimisation of intra-class variance or the maximisation of inter-class variance, widely known as Otsu’s Method [54]. Otsu’s method is well suited to bimodal distribution, in which two peaks in the image histogram are sharply separated [55]. However, this method performs well when the numbers of pixels in classes are similar [56], and it has the advantage of being easy to implement. Gustafsson et al. suggested a modified version of this approach for SPECT segmentation in which the histogram was not equidistantly binned, so that every voxel value in the initialisation VOI could serve as a threshold [48]. Extension to fixed threshold methods will be discussed in more detail in the next section concerning adaptive and automated thresholding.

### 4.3. Adaptive and Automated Thresholding

Fixing one global threshold value for the whole image is usually not sufficient for proper segmentation. To overcome this obstacle, the image background brightness needs to be corrected to have the same value over the whole image, or the threshold needs to be locally adapted to the local differences between object and background brightness. For nuclear medicine purposes, the second approach was selected, and adaptive thresholding methods were developed. 

Adaptive thresholding methods are usually based on series of phantom experiments to obtain curves corresponding to various image quality metrics, like source-to-background ratios, mean background intensity, or the spatial resolution (full width at half maximum (FWHM)) of the scanner for different object volumes. Based on these relationships, the optimal threshold value for a given object is found. The methodology assumes a priori knowledge of the object volume obtained from different modalities, like CT or MRI. Erdi et al. performed experiments for the PET imaging of lung lesions, with a set of curves for different source-to-background values to obtain the optimum threshold for a given object volume [57]. Daisne et al. suggested a simplified method with only one curve for a single threshold, independently of a priori knowledge of a lesion volume, for PET application in head and neck cancers [58]. Although, as was shown, the method is valid for small and poorly contrasted lesions (<4 mL), its accuracy strongly varies with the reconstruction algorithm, and PVE is not accounted for properly. Additionally, all these adaptive thresholding methods have the drawback of manual intervention, i.e., drawing the ROI, which is subjective and depends greatly on the ROI placement in an inhomogeneous background. This may lead to discrepancies. 

The iterative image thresholding method (ITM) proposed by Jentzen et al. for PET applications [59] was another milestone in the evolution of segmentation for functional tomography. Based on their work, a series of methods were developed to address the drawbacks of adaptive thresholding, also in SPECT applications [16,60]. 

The ITM is based on threshold–volume curves derived from phantom experiments to vary source-to-background ratios. In the next steps, the volume is estimated in an iterative process with the use of the above-mentioned calibrated curves. The method was validated with the use of a PET phantom of known sphere diameters and gold standard PET lesions of known volumes, assessed on CT images [59]. As a threshold, volume curves are derived for typical source-to-background (S/B) values, and a series of several curves is required to cover the observed range of changes in the S/B ratios. Based on the calibrated curves, a primary threshold value is obtained, which is approximated further in the next steps of the iterative algorithm to estimate the lesion volume. PVE and spillover were managed by the appropriate selection of activity concentration [59]. However, this method is prone to changes in the scanner, reconstruction algorithms, the greyscale of an image, and the radionuclide used, and it must be calibrated every time, because some of these parameters change. The method is also insufficient for lesions with effective diameter close to the scanner’s spatial resolution, due to the PVE. Another drawback is that ITM, along with other thresholding methods, is reliable only if the activity distribution is homogenous. 

Grimes et al. replaced the manual selection of the background regions with automatic selection with the use of an iterative methodology for SPECT. Additionally, they underlined the need for accurate activity estimates, along with volume determination with different thresholds, for the purpose of internal dose calculations [60]. The proposed ITM-based method replaced the manual delineation of background regions with the semi-automatic process of adequate background ROI selection. In the first step, an initial VOI is created that contains the object of interest and part of the surrounding background. In the next step, an initial volume assessment (VOI_1_) is made with the threshold set at 40% of the maximum count in the VOI. VOI_1_ is then used to calculate an initial estimate of the SBR (source-to-background ratio), where source activity (S_1_) is the average activity in the VOI_1_, and background activity (B_1_) is the average activity between VOI_1_ and VOI. Then, based on the calibration curves, new threshold values for volume (ThV) and activity (ThA) are estimated. ThV is then used to calculate a new S_2_ value in VOI, and a new B_2_ value is calculated in the same manner as previously but with the use of ThA. The process runs iteratively until a predefined limit of percentage difference between two adjacent SBRs is reached. The methodology was validated in a series of phantom experiments in which bottles of known different sizes and activities were inserted into large phantoms filled with background activity. Percentage errors between validation estimates of activity and volume and true values were calculated. Additionally, patient data were used for validation purposes. The authors demonstrated that two different thresholds are needed for the accurate determination of both the volume and activity concentration. The influence of reconstruction algorithms and image resolution was investigated; the general rule that an image with poorer spatial resolution revealed higher differences between two thresholds (volume threshold ThV and activity concentration threshold ThA) was confirmed. 

The ITM method evolved further, and a new recovering iterative thresholding method (RIThM) for SPECT was created with the implementation of the recovery coefficients of the imaging system. The RITHM method, developed by Pacilio et al., implements two types of curves, i.e., threshold–volume curves and recovery coefficient (RC) curves, to deal with partial volume effects. Additionally, Monte Carlo simulations have been tested as a means to obtain calibration datasets. Experiments showed that PVE strongly affects counts for volumes below 20 mL. However, because PVEs are dependent on the object size and spatial resolution, for a given volume, the associated RC curves were almost indistinguishable [16]. It is also important that the calibration results are strongly affected by reconstruction and filtering methods. As was proven, OSEM reconstruction for small objects needs to be well optimised. Again, it was shown that for volumes > 4 mL, the estimation and true value were in quite good agreement, with less than a 15% difference [16]. For smaller volumes (e.g., 4/3 of spatial resolution), the method was more stable in the iterative processes than ITM, but no good accuracy was yielded. For objects of volume > 20 mL, the results were similar to those of the ITM method, because the PVE did not influence the data. The group reported that Monte Carlo simulations were suitable to generate calibration data, which is an advantage in terms of radiation protection for staff. 

**Figure 1:**
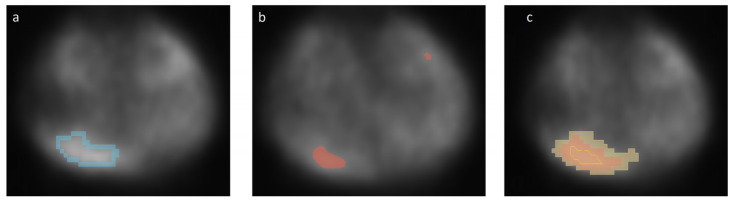
Comparison of the three main segmentation methods used in nuclear medicine. (**a**) Manual segmentation; (**b**) threshold segmentation; (**c**) region growing approach.

All the above-mentioned techniques are based on the assumption of geometric tumour shapes and uniform distribution of the activity in the structures and background, which is not the case in clinical practice. As such, there is a need to address these issues. SPECT experiments were conducted with the use of ^99m^Tc, not with ^177^Lu, mostly for radiation protection reasons. Figure 1 shows a comparison of the three main segmentation methods used in nuclear medicine. 

## 5. Region Growing Approach

This approach is an alternative to histogram-based methods that suffer from a lack of spatial information. Region growing aims to connect neighbouring regions with similar grey values. The procedure starts with a set of seeds points. Then, from each seed, a uniform, connected region grows out of that seed until the stopping criteria are met [56]. A pixel is assigned to the region based on the mean and standard deviation of the intensity criteria. A pixel can be added to the region if it has not been assigned to another region before, it is a neighbour of the region under consideration, and the region created by adding that pixel is uniform.

However, the method assumes that regions are nearly constant and slowly varying in image intensity. Additionally, the approach is strongly dependent on initialisation factors. As was shown, it works well in some PET applications, where the initialisation parameters were set properly and homogeneity assumptions were met. However, due to the “leakage” caused by PVE, poor spatial resolution, and motion artefacts that PET shares with SPECT, the method has shortcomings [43]. To address some of these problems, improvements have been proposed, like using a priori shape information (ROI manual definition) that is incorporated into the algorithm, and using the voxel of maximum intensity as a seed. When a sharp increase in volume is registered, it is assumed that the region transition from tumour to background has occurred, and the preliminary tumour boundary is set, just before this sharp increase. In the final step, a dual-front active contour technique is applied to refine the final contours. The method performs well for small lesions in PET images and has the advantage that it does not need calibration curves. Additionally, it is reproducible and independent of the initial manual ROI drawing [61]. 

Another method combines the region growing approach with the threshold-based algorithm, reducing the noise by assuming homogeneity and connectedness of the segmented regions to obtain more robust outcomes [43]. 

## 6. Boundary-Based Surface Adaptation—Parametrically Deformable Shape Models

In parametrically deformable shape models, the shape of a structure is obtained by deforming the surface for gradually increased Fourier orders. In a parametric representation of the surface, Fourier coefficients are optimised, taking into consideration objective functions. The set of Fourier coefficients represents the contour of the surface. Because a parametric representation requires only small number of parameters to model the surface area or curvature, it is a convenient way to analyse an object’s properties. It also enables the incorporation of some probabilistic information [62]. An objective function that, for the optimisation problem is the real-valued function, is a function whose value is minimised or maximized to find the optimal solution. To fit a model to the image data, optimisation of the model parameters is needed to optimise measure of fit; this allows us to distinguish the surface of an object from the background. The boundary strength (direction can also be used) computed from an image is a measure that indicates a change in some property that could be used for such a distinction. For images, such a measure could be a grey-level gradient. The magnitude is the strength of the boundary, and the direction is normal to the boundary [62]. For better results, it is suggested that the image be smoothed with a Gaussian filter to reduce noise effects. 

Gustafsson et al. suggested the use of Fourier surfaces in SPECT image segmentation in estimates of tumour volume and activity concentration in ^177^Lu-DOTATATE therapy [46]. The initial estimation is an ellipsoid that approximates an object, which is then optimised by changing the Fourier coefficient to find the resulting surface that reflects the SPECT image structure. First, the Fourier coefficients for the initial two (order surface and ellipsoid) are estimated. Then, the process of optimisation is implemented, and the number of Fourier orders is gradually increased to four after the convergence is reached for a given order. The strength of the edges may be measured with the use of an objective function in the form proposed by Floreby in [63].

Fourier functions are considered to be resistant to noise, which is an advantage with noisy SPECT images [48,64]. It may be assumed that the high-frequency modulation in surface descriptions of SPECT images reflects image noise. This is why only a few Fourier orders are used, which makes the methodology quite simple [62]. However, as was shown in the study [48], when compared to other methods like 42% threshold and Otsu (OM), Fourier surfaces (FS) performed better only for images acquired at 336 h post injection (p.i.) time point (volume error and DSC index). For the 24 h p.i. time point, OM and FS gave comparable results [48]. Simulated datasets were also investigated, and FS performed worse than other methods in that case. It should be underlined here that, as was shown in many studies, the overall performance of segmentation methods relies strongly on image characteristics like noise, spatial resolution, and image artefacts (especially edge artefacts, that can produce bias). Prior optimisation of the camera settings and the applied acquisition and reconstruction protocols are of a great importance; however, a better segmentation methodology that is more independent and robust is desired.

## 7. Stochastic and Learning-Based Methods 

Classification is a pattern recognition technique in the segmentation process in which labels are associated with regions to divide images by tissue type. This process requires prior knowledge about such labels; thus, pre-segmented images are needed as training data. During classification, the so-called feature space that is derived from an image is divided with the use of known labels. The feature space is built from the vectors of the features formed at each pixel. The pixel intensity and gradient of a given pixel may serve as such features. The nearest-neighbour classifier (NNC) is the simplest form of such a classifier, in which a pixel is classified to a given class based on its intensity. The classifier transfers the label from the training dataset for which the intensity is closest to that of a given pixel. Other examples of classifiers are k-nearest neighbours (k-NN), Fisher linear discriminant (FLD), support vector machine (SVM), nearest mean classifier (NMC) [56], and artificial neural network (ANN). These methods can transfer labels from training data to new data, but they do not take into account spatial information in the labelling process [43].

Clustering methods, in contrast, have the ability to incorporate spatial information in the process of image segmentation. The clustering process is similar to the classification, but clustering does not need training datasets. Its main disadvantage is that the appropriate number of clusters needs to be determined before the process starts [56]. Items with similar properties are gathered into local groups based on, e.g., intensity values or spatial location. Some examples of such methods are C-means, Fuzzy C-means (FCM), and the expectation-maximisation (EM) algorithm.

There are two categories of automated segmentation algorithms: supervised and unsupervised. Supervised algorithms are very efficient but require additional input data, usually ground truth images, to train the algorithm. Convolutional neural networks and recently exploited deep learning algorithms fall into this group. As they are trained on specific datasets, and they are prone to “unseen” domains, leading to poor outcomes in such cases. Conversely, unsupervised methods are more robust at processing “unseen” domains, and they are useful in situations when there is a lack of good quality data for training [65]. 

**Figure 2:**
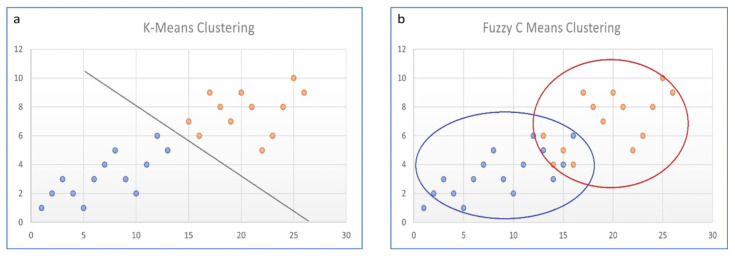
Hard vs. soft classifiers (**a**) k-means clustering (hard); (**b**) Fuzzy C-means clustering (soft).

Because nuclear medicine images suffer from low resolution and partial volume effects, fuzziness occurs; so-called “hard” segmentation algorithms that segment the voxel to only one particular class are usually not sufficient. Hard algorithms are, e.g., K-means or active contour. On the other hand, in so-called “soft” or “fuzzy” algorithms, each data point has an assigned probability belonging to probable, multiple classes (see Figure 2). 

Fuzzy C-means (FCM) is one such soft algorithm of known suitability for nuclear medicine low-resolution images. This method is simple, robust, and effective; however, it does not take spatial context information into account, which may lead to artefacts and noise in segmentation [65].

### 7.1. Fuzzy C-Means

Fuzzy C-means, due to its convenient implementation, is widely used in nuclear medicine, mostly for PET applications, but also SPECT applications. Several approaches have been used to overcome the drawback of a lack of spatial context information that incorporates voxel position with regard to the neighbourhood [66], i.e., a spatial penalty function that allows spatially smooth membership functions to be obtained [67]. 

The Fuzzy C-means algorithm partitions a collection of n elements into *c* fuzzy clusters based on a given set of criteria. Each observation belongs to the cluster with the nearest mean (cluster centre or cluster centroid). Firstly, the mean of all voxel intensities is calculated, and then voxels with intensities lower than the mean are assigned to the first cluster and are taken out of the image data. Secondly, the new mean of the remaining voxels is calculated, and the process is repeated to obtain the second cluster. The whole procedure is iteratively repeated for a given number of clusters. When the clustering is finished, the membership function is assigned for each voxel in the clusters. An algorithm returns a list of c cluster centres and a partition matrix, in which each element is given a degree to which the voxel belongs to cluster *c*. The objective function of the FCM algorithm is given by

(10) $$J_{FCM}=\sum_{k=1}^{n}\sum_{i=1}^{c}{\left(u_{ik}\right)}^{m\;}{\left\|x_{k}-v_{i}\right\|}^{2}\;\mathrm{subject}\;\mathrm{to}\;\sum_{i=1}^{c}u_{ik}=1$$

where *X* = {*x*_1_, *x*_2_,… *x*_n_} is the data matrix of dimension *p* × *n*, *p* refers to feature data vectors *x_k_*, *n* is their number, *V* = (*v*_1_, *v*_2_,… *v_c_*) is a vector of unknown clusters centres (prototypes), *u_ik_* is the membership function of the *k*-th vector to the *i*-th cluster, and *m* is a factor to adjust the membership degree weighting effect (that controls the fuzzy overlap between clusters), usually set as 2 [66,68].

(11) $$v_{i}=\frac{\sum_{k=1}^{n}u_{ik}^{m}x_{k}}{\sum_{k=1}^{n}u_{ik}^{m}}\;,1\leq i\leq c$$

(12) $$u_{ik}=\frac{1}{\sum_{j=1}^{c}{\left(\frac{\left\|x_{k}-v_{i}\right\|}{\left\|x_{k}-v_{j}\right\|}\right)}^{2/(m-1)}}\;,1\leq i\leq c\;,1\leq k\leq n$$

The objective function iteratively estimates cluster centres and membership functions until FCM reaches a convergence. The FCM algorithm aims to minimise objective function converging to local minima or saddle points, which implies a set of optimum parameters. Several upgrades to the standard FCM algorithm have been proposed to overcome its limitations, mostly in PET applications [43,66,67]. Pham et al. incorporated spatial constraints to the objective function in the FCM model, which makes it possible to label pixels with the influence of the neighbouring pixel labels [67,69]. The method makes use of Markov Random Field theory. Chen et al. suggested a method [65] to combine the properties of intensity clustering with a deep learning approach. Their objective function is based on that of the traditional FCM algorithm, in which fuzziness is controlled by a user-defined hyperparameter. 

### 7.2. AI Deep Learning Algorithms

In recent years, AI-based solutions to segmentation tasks have been proposed to overcome the shortcomings of the various methods. In machine learning approaches, artificial intelligence (AI) tends to recognise a pattern automatically and extract a desirable representation from raw data. The deep learning (DL) approach is a subtype of the machine learning approach, in which a feature is extracted, selected, and classified automatically in a single step [70]. There are two main types of DL algorithms used in nuclear medicine: convolutional neural networks (CNNs) and generative adversarial networks (GANs) [70].

#### 7.2.1. Convolutional Neural Networks (CNNs/ConvNet)

**Figure 3:**
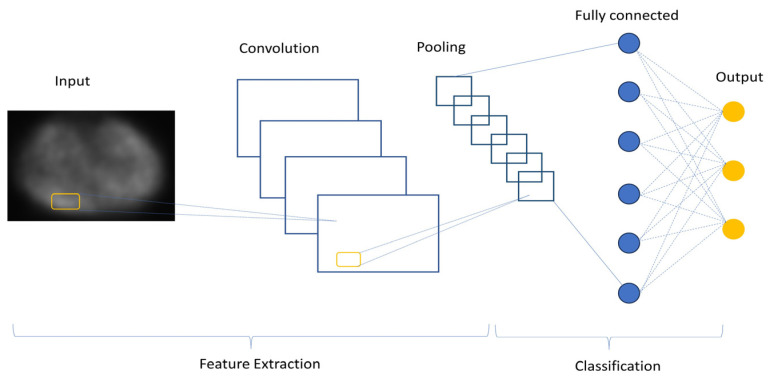
Convolutional neural network architecture.

The convolutional encoder–decoder (CED) deep learning architecture has been introduced to the field of nuclear medicine, whereby input images are converted to feature vectors, that are further converted to target images. CNNs have many variations in terms of their architecture, but there are three major components that can be distinguished: input layers, feature extraction (learning) layers, and classification layers (Figure 3). The input layer accepts the 3-dimentional inputs, with the depth representing RGB colour channels. In the feature extraction layers, one can find a repeating schema of a convolutional and pooling layers, the first of which is a hidden layer that applies a convolution filter to the input data to obtain an activation map (feature map), which, in turn, learns the patterns to extract the features. Convolutional layers are the core building blocks of CNN architecture. ConvNet is a weakly-supervised method that does not require extensive training datasets.

#### 7.2.2. Generative Adversarial Networks (GANs)

These kinds of neural networks are able to synthesise new images based on training images. They use an unsupervised learning approach to train two models in parallel. They consist of two components: a generator, which is usually a CNN network, and a discriminator, i.e., a classifier that is able to differentiate ground truth data from synthetic data [70]. The training runs until the discriminator is not able to find the differences between the real and synthetic data. This approach has been implemented in SPECT and PET applications for image denoising tasks and in low-dose and fast SPECT imaging studies [71].

## 8. Discussion

This review provides an overview of SPECT segmentation approaches, with particular emphasis on applications in ^177^Lu-DOTATATE therapy. However, very few studies have covered this important topic. Additionally, some PET applications have been reviewed that could be of interest for SPECT applications, due to the similarities in the image characteristics in the two approaches. Little is known about the performance of certain algorithms with ^177^Lu as a radiotracer, for which the activity concentration and its location are different, because little research has been carried out to date [35,48,72]. A summary of segmentation methods and their results is shown in Table 1. 

The main limitations in SPECT segmentation are image degrading factors, which undermine the accuracy of the proposed methods. Inherited degrading factors, like high noise levels, poor spatial resolution, partial volume effects, and spill-out effects, lead to mispositioning of the activity in the image and, hence, underestimates of the activity concentrations in volumes with high activity. This leads to further inaccuracies in internal dose assessments. However, huge effort has been made to overcome these obstacles and to find a reasonable solution for segmentation tasks. The vast majority of methods, from fixed thresholding and adaptive thresholding to boundary based, region growing, and stochastic methods, as well as learning based methods, which are being extensively researched at present, were proposed to resolve problems associated with the segmentation task.

Some of the proposed methods are time consuming and require highly trained staff, meaning that they can only be applied with success in highly specialised centres. For manual delineation to obtain ground truth, user-drawn volume of interest, and the definition of background regions, and the manual selection of seeds to start the algorithm or set the starting parameters, the implantation of almost all algorithms requires some level of user interaction and, in many cases, the incorporation of high-level expertise. Additionally, validation in clinical settings is a challenging issue [73]. Most of these methods are strongly dependent on the device, acquisition protocols, and reconstruction methods used in the clinic, and as such, they are poorly transferable to other clinics without proper and time-consuming validation. To address this, the American Association of Physicists in Medicine task group No. 211 suggested, for the purposes of the validation of PET segmentation algorithms, the following as performance criteria: accuracy, precision (reproducibility and repeatability), efficiency, and robustness [74]. It is also advisable to use patient datasets along with the phantom experiments.

As is clear from the reviewed publications, methods of SPECT segmentation, similarly to PET, are advancing into fully automated processes with the use of artificial intelligence. DL methods are robust and efficient, but their “black box” nature reveals a strong need for the standardisation of segmentation methods and a proper validation framework, preferably with the use of publicly available datasets. 

## 9. Future Directions

SPECT image segmentation is of great importance in molecular radiotherapy dose assessments. This review has revealed that this challenging task has been investigated thoroughly by only a few research groups. However, with the implementation of the BSS Directive in the European Union and FDA and EMA approvals for Luthatera^®^, the need for patient-specific dosimetry in ^177^Lu-DOTATATE therapy is growing, and with it, the importance of robust and effective segmentation. This investigation has shown that there are some segmentation methodologies with different degrees of complexity that could be used depending on the capacity of a given centre. Some of these methods have also been implemented in commercially available software. Nevertheless, there is still room for further investigation in this field.

## Figures and Tables

**Table 1:** Summary of segmentation methods for SPECT imaging.

Lp	Reference	Radionuclide	Methods	Validation Phantom/Data Sets	Results
1.	Dewaraja et al. [72]	^177^Lu	Lesions: manual segmentation Organs: on CT with higher mAs, at a reference point, CNN algorithms	20 patient data sets (77 lesion)	CNN defined kidneys with manual adjustment to manual segmentation— DSC (0.91–0.94)
2.	Pacillo et al. [16]	^99m^Tc	Lesions: RIThM—upgraded ITM with RC (ITM as a comparator)	Jaszczak phantom + test objects Test images (hot spheres, Zubal head phantom simulated with SIMIND code) 3 brain metastasis + 2 gliomas	Accuracy level: 10% (for vol 20–110 mL); Difference between estimated and true volume: 15% (vol > 4 mL) for ITM and RIThM
3.	Gustafsson et al. [48]	^177^Lu	Lesions: Fixed threshold 42%—FT Otsu Method—OM Fourier Surfaces—FS	XCAT phantoms, MC simulated SPECT images in different time points	Volume and activity concentration root-mean-square error < 15% for tumours > 10 mL for OM and FS (FT worse); FS robust to noise, better for 366 h p.i. time point
4.	Chen et al. [65]	^99m^Tc	Bone metastasis: DL, CNN with FCM and novel loss functions	Clinical datasets Simulated SPECT/CT images XCAT	DSC 0.75 and 0.74 for lesions and bone segmentation on SPECT images, respectively; DSC of 0.79 bone segmentation on CT scans
5.	Grimes et al. [60]	^99m^Tc	Organs: (kidneys, liver, spleen, thyroid) and lesions: Iterative adaptive thresholding (ThV and ThA) Semiautomatic background selection	Phantoms with 20 different inserts 13 patient studies	Object volume and activity estimation separately
6.	Uribe et al. [35]	^177^Lu	FT of 40%; CT-based segmentation; Iterative Adaptive Dual Thresholding (IADT)	Phantoms—hot objects in warm water	Volumes > 34 mL—10% error (percent difference between experimental and true activities)

## Data Availability

No new data were created or analyzed in this study. Data sharing is not applicable to this article.
